# Antiviral Effect of Epigallocatechin Gallate via Impairing Porcine Circovirus Type 2 Attachment to Host Cell Receptor

**DOI:** 10.3390/v12020176

**Published:** 2020-02-04

**Authors:** Jiarong Li, Dongfeng Song, Shengnan Wang, Yadong Dai, Jiyong Zhou, Jinyan Gu

**Affiliations:** 1 Institute of Immunology, College of Veterinary Medicine, Nanjing Agricultural University, Nanjing 210095, China; 2015207025@njau.edu.cn (J.L.); 2017107087@njau.edu.cn (D.S.); wangshengnanwsnj@163.com (S.W.); 2 MOA Key Laboratory of Animal Virology, Institute of Preventive Veterinary Sciences and Department of Veterinary Medicine, Zhejiang University, Hangzhou 310058, China; 11817028@zju.edu.cn (Y.D.); jyzhou@zju.edu.cn (J.Z.); 3 Center of Veterinary Medical Sciences, Zhejiang University, Hangzhou 310058, China

**Keywords:** Porcine circovirus type 2, epigallocatechin gallate, heparan sulfate, antiviral effect, virus attachment

## Abstract

The green tea catechin epigallocatechin gallate (EGCG) exhibits antiviral activity against various viruses. Whether EGCG also inhibits the infectivity of circovirus remains unclear. In this study, we demonstrated the antiviral effect of EGCG on porcine circovirus type 2 (PCV2). EGCG targets PCV2 virions directly and blocks the attachment of virions to host cells. The microscale thermophoresis assay showed EGCG could interact with PCV2 capsid protein in vitro with considerable affinity (Kd = 98.03 ± 4.76 μM), thereby interfering with the binding of the capsid to the cell surface receptor heparan sulfate. The molecular docking analysis of capsid–EGCG interaction identified the key amino acids which formed the binding pocket accommodating EGCG. Amino acids ARG51, ASP70, ARG73 and ASP78 of capsid were found to be critical for maintaining the binding, and the arginine residues were also essential for the electrostatic interaction with heparan sulfate. The rescued mutant viruses also confirm the importance of the key amino acids of the capsid to the antiviral effect of EGCG. Our findings suggest that catechins could act as anti-infective agents against circovirus invasion, as well as provide the basic information for the development and synthesis of structure-based anti-circovirus drugs.

## 1. Introduction

Circovirus is one of the smallest non-enveloped viruses with a single-stranded circular genomic DNA and classified in the genus *Circovirus* of family *Circoviridae* [1]. It infects various species ranging from plants to animals with different pathogenicities [2]. Porcine circovirus type 2 (PCV2) is a prototype circovirus that causes significant morbidity and mortality in swine [3,4,5], and is associated with different syndromes, such as the post-weaning multisystemic wasting syndrome [6,7] and reproductive disorder [8]. The genome of PCV2 contains two major open reading frames (ORFs). *ORF1* encodes the replicase protein (Rep) involved in rolling-circle viral DNA replication, while *ORF2* encodes the unique viral structural protein capsid [9], which is involved in diverse and essential biological events during virus infection, such as virion attachment [10].

The primary attachment of the non-enveloped virus on the host cells is based on a low-affinity interaction between viral structural protein and cell surface glycosaminoglycans (GAGs) receptors [11,12]. GAGs include heparan sulfate (HS), keratan sulfate (KS), and chondroitin sulfate (CS), which are characterized by disaccharide units forming blocks of polysaccharides [13]. Among these GAGs, HS is the most common polysaccharide molecule utilized by viruses for attachment to cell surfaces, such as the hepatitis C virus [14], herpes simplex virus 1 [15], human enterovirus 71 [16] and rabies virus [17]. PCV2 has also been verified to use cell surface HS as attachment receptor, and adopts a distinct non-symmetrical receptor distributed pattern on the virion with a multitude of weak HS binding sites [10,18]. Chemical molecules acting as receptor mimics that interfere with HS-virion interactions might exhibit antiviral activities [19]. However, there are very few reports of PCV2 infection inhibition available for pertaining to such natural small molecule compounds.

The green tea catechins (GTCs) are polyphenolic compounds extracted from the leaves of *Camellia sinensis* [20], and have shown numerous beneficial properties, such as anticancer activity [21], anti-inflammatory ability [22], antioxidative properties [23], antibacterial function [24] and antiviral effects [25]. GTCs are predominantly comprised of four constituents: epicatechin (EC), epigallocatechin, epicatechin gallate, and epigallocatechin gallate (EGCG) [23], among which the EGCG is the major component of GTCs accounting for approximately 59% of the total polyphenols, and it is also the most complicated and principal constituent, possessing the activity against infection of several viruses, such as human immunodeficiency virus (HIV) [26], influenza A virus [27], Zika virus [28], herpes simplex viruses 1 [29], and hepatitis C virus [30]. The mechanisms of EGCG antiviral activity are complicated and diverse, depending on the specific virus infection process or host cell response, one of which is that EGCG interacts with viral structural proteins, consequently blocking the latter from recognizing or binding with cellular receptors. In previous reports, EGCG has been shown to interact with the influenza A virus hemagglutinin, disrupting the binding of envelope glycoprotein to cell surface sialic acid receptor [25]. EGCG inhibited the interaction of HIV envelope glycoprotein gp120 with the cellular CD4 molecule [31]. Regarding the GAGs receptors, EGCG could bind to the herpes simplex virus-1 glycoprotein gB and gD, which should interact with HS and 3-O-sulfated HS of cellular glycans, respectively [29,32].

In previous studies, there have been no reports of catechins inhibiting circovirus invasion, we aimed to investigate the effect of EGCG on PCV2 infection.

## 2. Materials and Methods

### 2.1. Cells and Virus

PCV-free Porcine Kidney 15 (PK15) (BH0370, ATCC, Manassas, VA, USA) cells were supplied by China Institute of Veterinary Drugs Control and cultured in Opti-MEM (11095-072, Thermo Fisher, Waltham, MA, USA) with 10% fetal bovine serum (10099-141-FBS, Thermo Fisher). PCV2 strain HZ0201 (GenBank accession no. AY188355.1, 10^6.5^TCID_50_/mL) was propagated in PK15 cells [33]. PCV2 virions were enriched using ultra-centrifugation.

### 2.2. Reagent and Antibodies

Epigallocatechin gallate (EGCG, 99.91% purity, HY-13653, Medchemexpress, Monmouth, NJ, USA), epicatechin (EC, 99.00% purity, HY-N0001, Medchemexpress) and heparin (99.00% purity, H3393, Sigma-Aldrich, St. Louis, MO, USA) were dissolved into ddH2O at a concentration of 50 mM for storage. The monoclonal antibody (mAb) 5E11 against PCV2 capsid was generated in our laboratory [34]. The fluorescein isothiocyanate (FITC) conjugated goat anti-mouse IgG (ab6785, Abcam, Cambridge, MA, USA), horse radish peroxidase (HRP) conjugated goat anti-mouse IgG (074-1802, KPL, Milford, MA, USA) and Mouse IgG1 Isotype Control (564416, BD Horizon™, San Jose, CA, USA) were purchased from Abcam, Kirkegaard & Perry Laboratories and BD Biosciences, respectively.

### 2.3. Cytotoxicity Assay

The cytotoxicity assay of compounds was performed using enhanced CCK8 kit (C0014, Beyotime, Shanghai, China) according to the standard instructions. PK-15 cells seeded into 96-well plate was treated with different concentrations of EGCG or EC for 12 h. Subsequently, 10 μL CCK8 reagent per well was added into the culture medium and after 1 h of incubation, absorbance value (A450) was measured by a spectrophotometer. The cell viability was calculated as (A450_compound_/A450_mock_) × 100%.

### 2.4. Determination of Virus Titer

PK-15 cells seeded in the 96-well plates were added with 10-fold serially diluted virus samples. At 96 hpi (hours post-infection) the virus titers were determined by indirect immunofluorescent assay (IFA) with mAb 5E11 against PCV2 capsid and FITC-conjugated goat anti-mouse IgG. The 50% tissue culture infective dose (TCID_50_) was calculated according to the Reed-Muench method. The multiplicity of infection (MOI) was defined as the number of plaques forming units (PFUs) computed as PFU = 0.7 × TCID_50_.

### 2.5. Calculation of EC_50_

EGCG was serially diluted to 100, 50, 25, 12.5, 6.25 and 3.125 μM, and was added to PK-15 cells which were infected with PCV2 at MOI = 1.0. EGCG was present during whole PCV2 infection. The infected cells without EGCG treatment was set as mock control. TCID_50_ was determined at 72 hpi and the values of inhibition were calculated as 1 − TCID_50(compound)_/TCID_50(mock)_ × 100%. The values of inhibition and EGCG concentration were used to establish a dose response curve and further calculate the EC_50_ (concentration for 50% of maximal effect).

### 2.6. Infectivity Assay

PCV2 samples were pretreated with chemical compound at 37 °C for 10 min, then inoculated into near-confluent PK-15 cell monolayers at a MOI = 1.0 at 37 °C. Virus inoculum was removed after 1 h and the infected cells were maintained in medium containing the compound. Virus infectivity was assessed by titer determination at various time points. Meanwhile, at 72 hpi the virions in the infected cells were detected by IFA with mAb 5E11 FITC-conjugated goat anti-mouse IgG; expression level of the PCV2 capsid protein was measured by western immunoblotting using 5E11 antibody and HRP- goat anti-mouse IgG; copy numbers of the viral genome were determined with quantitative real-time PCR (qRT-PCR) with forward primer (TACTGCTGTGAGTACCTTGTTGGA) and reverse primer (TCTGCATTTTCCCGCTCACT) targeted to the 359~483 bp sequence (124 bps) of PCV2 *Rep* gene, and the probe was designed as ‘FAM-AGTCTGGTGACCGTTGTTGCAGAGCAGCAC-BHQ’. The pMD18-*Rep* plasmid was serially diluted 10-fold to 10^3^~10^7^ to generate the standard curve and the copy numbers of samples were quantified using absolute quantification method [35].

### 2.7. Time of Addition and Dilution Experiment

PK-15 cell was pre-exposed to EGCG or Opti-MEM medium at 37 °C for 60 min, then washed and infected with PCV2 at a MOI = 1.0 at 37 °C for 1 h before the inocula were removed, the samples were maintained without EGCG and the infectivity was assessed at 72 hpi. Alternatively, the PCV2 was premixed with EGCG under the same condition before being added to cells, the samples were maintained and determined with same way. For post-treatment samples, cells were first inoculated with PCV2 at a MOI = 1.0 at 37 °C, followed by 100 μM EGCG treatment maintained for 4 h, 12 h and 24 h, respectively, the samples were tested at 72 hpi. For dilution experiments, approximately MOI = 10.0 of enriched virions were pretreated with 100 μM EGCG for 60 min at 37 °C, and then diluted 10-fold before being added to cell monolayers, infected cell samples were maintained without EGCG after the inocula were removed 1 h. Infectivity of samples was assessed at 72 hpi.

### 2.8. Binding Assay and Flow Cytometry

PCV2 virions enriched with ultra-centrifugation were added to PK-15 cells with MOI = 5.0 in the presence of 100 μM EGCG. After binding for 1 h or 4 h at 4 °C, the mixture was then washed off and the cells were harvested. The amount of the attached virions was determined using qPCR. The ratio of attached cells was measured by flow cytometry, the trypsin digested PK15 cells were resuspended and incubated with the mAb 5E11 for 1.5 h at 37 °C, followed by staining with FITC-conjugated goat anti-mouse IgG for 1 h, mouse IgG1 was used as isotype control, finally the samples were measured by BD Accuri™ C6 flow cytometer (BD bioscience, San Jose, CA, USA) and the results were analyzed by FlowJo X software (Version 10.0.7, FlowJo, San Jose, CA, USA ).

### 2.9. Recombinant Protein Expression and Purification

The engineered *dcapsid* (capsid with N-terminal nuclear localization sequence deleted) gene of PCV2 strain HZ0201 was cloned and inserted into pET28 vector encoded with a hexahistidine tag at the N-terminus. The dcapsid mutants with alanine substituted for specific amino acids were constructed using site-specific mutagenesis PCR technology. The vectors were transformed into *Escherichia coli* BL21 (DE3) and the protein expression was induced using 1 mM Isopropyl β-d-1-Thiogalactopyranoside at 16 °C overnight. The recombinant proteins were purified using Ni-NTA super-flow matrix (30410, Qiagen, Germantown, MD, USA), followed by PD10 column (17-0851-01, GE healthcare, Los Angeles, CA, USA) to remove the excess imidazole. The concentration of recombinant protein was measured using the BCA method and the protein samples were stored at −80 °C.

### 2.10. Heparin Column Chromatography

The purified recombinant PCV2 dcapsid were bound to a 5-mL HiTrap™ Heparin-Sepharose HP Column (17040701, GE Healthcare, Los Angeles, CA, USA) in loading buffer (10 mM sodium phosphate, 0.3 M NaCl, pH 7.4) at a flow rate of 1 mL per minute. The column was washed with 10 mL loading buffer to remove excess molecules, and thereafter eluted with soluble heparin, EGCG or EC competitor in loading buffer. The remaining bound protein was ultimately eluted with 3 M NaCl in loading buffer because the electrostatic interactions between Heparin-Sepharose and heparin-binding proteins could be disrupted by high salt concentrations. The original and eluted fractions were diluted to equal volumes, and analyzed using Western immunoblotting with the mAb 5E11 and HRP- goat anti-mouse IgG. The intensities of the immunoreactive protein bands were measured using Image J software (Version 1.52 g, ImageJ, Bethesda, MD, USA).

### 2.11. Microscale Thermophoresis Assay

The purified dcapsid proteins were labelled with the Cy5 fluorophore based on the chemical reaction between the ^108^cysteine of capsid and Cy5-maleimide (PA25031, GE Healthcare Life Sciences). The labelled molecules were separated from the unreacted and excess dyes with a PD Mini-Trap G-25 desalting column, and mixed at 10–50 nM concentration with 2 fold serially dilutions of EGCG, EC or heparin in reaction buffer (150 mM NaCl, 50 mM Tris-HCl, 10 mM MgCl_2_, 0.05% Tween-20, pH 7.4). The binding reactions were fixed into equal volume and incubated at room temperature for 15 min. The mixtures were then enclosed in standard pretreated glass capillaries and analyzed with MST machine (Monolith NT.115, NanoTemper Technologies, Munich, Bavaria, Germany), and the Kd values of each reaction were computed by NanoTemper Analysis software (Version 2.1, NanoTemper Technologies, Munich, Bavaria, Germany).

### 2.12. Construction of PCV2 Infectious Clone and Virus Rescue

The whole genome of PCV2 strain HZ0201 was cloned and inserted into pMD18-T vector (Takara, Tokyo, Japan). Site-specific mutagenesis was performed using PCR to construct the mutants. The linear genomes of the WT and mutants were extracted, digested by *Eco* RI restriction enzyme (NEB), cyclized by T4 DNA ligase (Takara), and transfected into PK15 cells for virus rescue with the jetPRIME^®^ in vitro transfection reagent (Polyplus-transfection, New York, NY, USA). The transfected cells were maintained for 72 h, then continuously passaged and cultured. The rescued virus samples were harvested and inoculated to PK15 cells and the infectivity was evaluated with immunoblotting in an indirect immunofluorescence assay. The virus titers were calculated according to the Reed-Muench method.

### 2.13. Statistical Analysis

All results were presented as mean ± standard deviation (SD) based on three independent experiments. Significant differences between experimental and control groups were analyzed using Student’s *t*-test. The differences were considered significant or highly significant at *p*-values < 0.05 and < 0.01, respectively.

## 3. Results

### 3.1. EGCG Inhibits the Infectivity of PCV2

**Figure 1:**
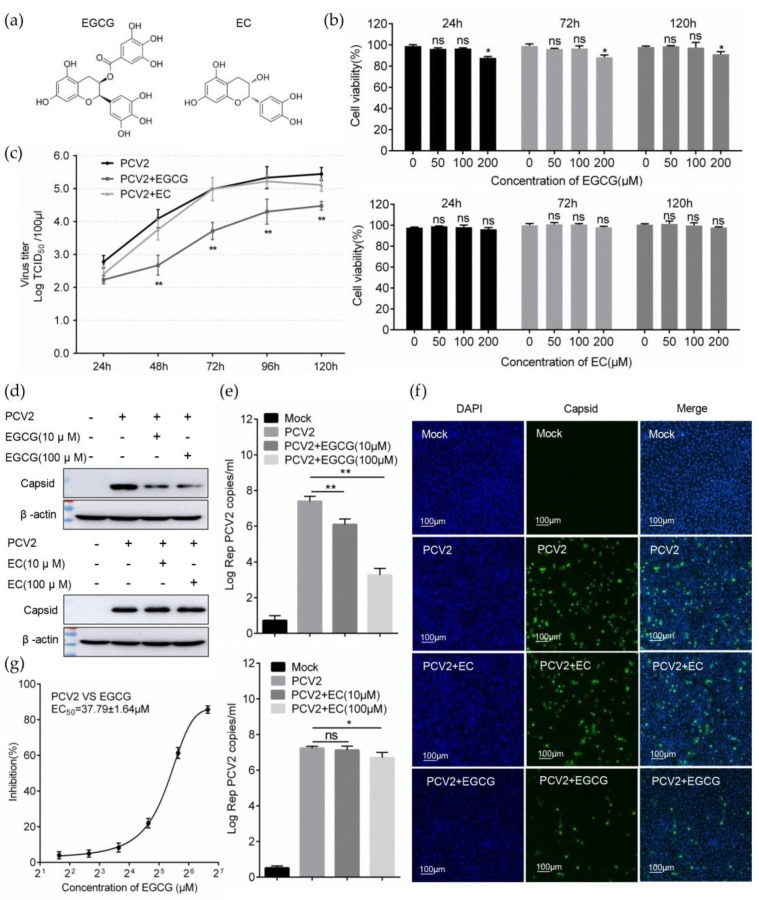
EGCG exhibits an anti-viral effect on PCV2 infection. (**a**) Molecular structure of EGCG and EC. (**b**) Cytotoxicity assay of soluble EGCG and EC. PK-15 cells were added with a concentration of 0, 10, 50, 100 and 200 μM EGCG or EC and cultured for 24 h, 72 h and 120 h, respectively. Cell vitality was detected by CCK8 assay and calculated as (A450_compound_ / A450_mock_) × 100%. (**c**) One-step growth curve of PCV2 with EGCG treatment. PK-15 cells were inoculated with PCV2 at MOI = 1.0 and cultured with 100 μM EGCG or EC treatment. TCID_50_ at various time points were determined by IFA and calculated according to the Reed-Muench method. (**d**–**f**) EGCG inhibited the infectivity of PCV2. PK-15 cells were inoculated with PCV2 at MOI=1.0 with EGCG or EC addition. (**d**) The expression inhibition of PCV2 capsid protein was detected by immunoblotting at 72 hpi. (**e**) PCV2 genome copies were measured by real-time PCR. (**f**) Indirect IFA detection of PCV2 infected cells. (**g**) Dose response curve and EC_50_ of the EGCG’s antiviral effect on PCV2. The mean ± SD of three independent experiments was compared using Student’s *t*-test (n.s., not significant; * *p* < 0.05; ** *p* < 0.01).

To assess the effect of EGCG on the infectivity of PCV2, the cytotoxicity on PK15 cells of the compounds was first examined using the CCK8 assay. The results showed that EGCG at a concentration of 100 μM did not generate significant cytotoxicity to PK-15 cells with a duration time of 24 h, 72 h, and 120 h, and EGCG at a concentration of 200 μM could produce cytotoxicity to cells, which limited the maximum concentration of EGCG at 100 μM (Figure 1b). The infectivity of PCV2 was significantly inhibited at the 100 μM EGCG treatment during the whole infective process, the virus titer of PCV2 infected cells with EGCG treatment showed a significant decrease at various time points compared to that without EGCG treatment (Figure 1c). Inhibition of PCV2 infectivity was also manifested by reduced level of viral capsid protein expression measured by immunoblotting (Figure 1d), and the reduction of viral genome copy number detected by qPCR (Figure 1e) at 72 hpi with the 100 μM EGCG treatment. The inhibitory effect of PCV2 infection was still detectable for the 10 μM EGCG treatment (Figure 1d,e). Furthermore, the cells infected with PCV2 at MOI = 1.0 were detected with IFA, which showed that the quantity of the PCV2 infected cells with the 100 μM EGCG treatment was obviously lower than that without EGCG treatment (Figure 1f). The original catechin EC displayed no considerable anti-infective effect on PCV2 under similar treatment conditions (Figure 1c–f). In addition, the EC_50_ of the antiviral effect of EGCG on PCV2 was further determined to be 37.79 ± 1.64 μM, which was consistent with its inhibitory concentration of 100 μM. Accordingly, EGCG could exert a prominent antiviral effect against the PCV2 infection.

### 3.2. EGCG Exerts the Antiviral Effect via Directly Targeting Virions

**Figure 2:**
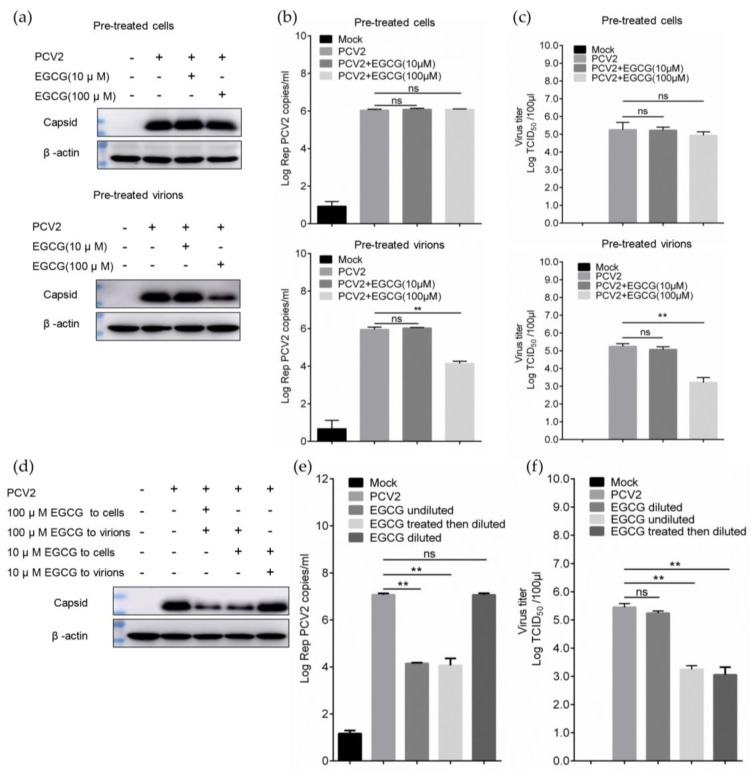
EGCG inhibits the PCV2 infectivity by directly targeting virions. (**a**–**c**) Cells were pre-exposed to 10 μM or 100 μM EGCG for 60 min at 37 °C, then infected with PCV2 at MOI = 1.0 for 1 h, alternatively, virus was pre-mixed with EGCG under the same condition before being added to the cells, then the cells were cultured without EGCG. At 72 hpi PCV2 infectivity was assessed by viral protein expression (**a**), viral genome copies (**b**), and virus titers (**c**). (**d**–**f**) Virions were pretreated with 100 μM EGCG and thereafter diluted 10-fold prior to infecting cells, which were cultured without EGCG and tested with immunoblotting (**d**), qPCR (**e**), and virus titers determination (**f**). Three independent experiments were performed and the results were compared using Student’s *t*-test (n.s., not significant; ** *p* < 0.01).

The antiviral effect of EGCG might be exerted by targeting PCV2 virions, such as the effect on certain viral nucleic acids and proteins, or targeting host cells [23]. To assess this issue, the time of addition assay was preformed, the cells were pretreated with EGCG and then inoculated with PCV2, or the PCV2 was premixed with EGCG before seeding to cells. Surprisingly, the inhibitory effect could not be detected with immunoblotting in the pre-treated cells group (Figure 2a), which was consistent with results of viral nucleic acid and titer determination (Figure 2b,c). Meanwhile, the prominent antiviral effect could be displayed in the pre-treated virions group with high EGCG concentration (100 μM), but non-sensitive to the low EGCG concentration (10 μM) pretreatment (Figure 2a–c), suggesting the compound might interact with virions. To rule out the possibility that the EGCG might also act on some cellular factors activated only in the presence of virions or some intracellular signaling pathways induced by the virus stimulation, we performed the dilution assay. Briefly, virus samples were pretreated at a high EGCG concentration (100 μM), and thereafter diluted 10-fold before being inoculated on the cells, ensuring the cells were treated with compound at 10-fold lower concentrations (10 μM) than the viral particles; in such a way, the inhibitory phenomenon would disappear if the compound really targeted host cells only in the presence of virions. However, the antiviral effect could still be detected with immunoblotting, qPCR and virus titer determination (Figure 2d–f). Considering that the pretreatment of cells with 10 μM EGCG could not trigger antiviral activity (Figure 2a–c), this inhibition was attributed to the effect of 100 μM EGCG on virions, further supporting that EGCG performed an antiviral effect via directly targeting virions.

### 3.3. EGCG Impairs the Attachment of PCV2 to Host Cells

**Figure 3:**
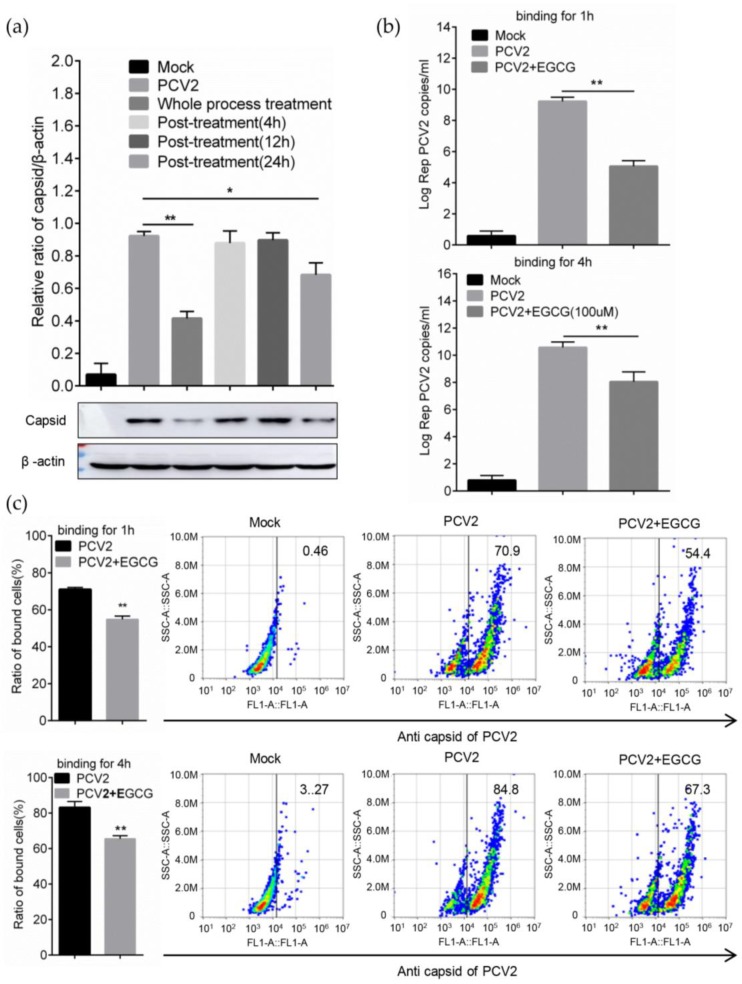
EGCG prevents PCV2 virions binding to host cells. (**a**) Cells were inoculated with PCV2 at MOI = 1.0 with 100 μM EGCG treatment during all the phases, or followed by post-treatment of EGCG for 4 h, 12 h and 24 h, respectively, after which the cells were maintained without EGCG until determined with immunoblotting at 72 hpi. (**b**,**c**) Virus was added to PK-15 cells at MOI = 5.0 in the presence of 100 μM EGCG. After the binding for 1 h or 4 h at 4 °C. the genome copy numbers of the attached virions were determined using the qPCR method (**b**); PCV2-attached cells were measured by flow cytometry (**c**). Mean ± SD of three independent experiments were shown and Student’s *t*-test was conducted for statistical analysis (* *p* < 0.05; ** *p* < 0.01).

To investigate which stage of the EGCG antiviral effect could be active during the PCV2 infective process, EGCG was added post-infection and incubated for various time. The results showed that compared with of the EGCG whole process treatment PCV2 infection, a significant inhibitory effect could not be detected with treatment for 4 h or 12 h after PCV2 infection, only a slight effect was observed when the post treatment duration reached 24 h (Figure 3a), indicating that EGCG might work at the early stage of infection course. Thereby, the binding assay was performed, the virions were added to the cell surface at 4 °C to achieve the binding event for 1 h, and the results indicated that bound PCV2 particles would decrease significantly in cells with the EGCG treatment measured by qRT-PCR (Figure 3b). The cells attached by the virions could be detected by flow cytometry [17], and its proportion also dropped from ~70% to ~55% in cells with the EGCG treatment for 1 h (Figure 3c). This tendency was also detectable when the binding time was extended to 4 h to maximize virions binding, and the proportion of PCV2-attached cells reduced from about 85% to about 67% (Figure 3b,c). Taken together, these findings demonstrated that EGCG inhibited the binding of PCV2 to host cells, but exerted no distinct effect on downstream infection.

### 3.4. EGCG Competitively Inhibits the Interaction between Capsid and Heparan Sulfate

**Figure 4:**
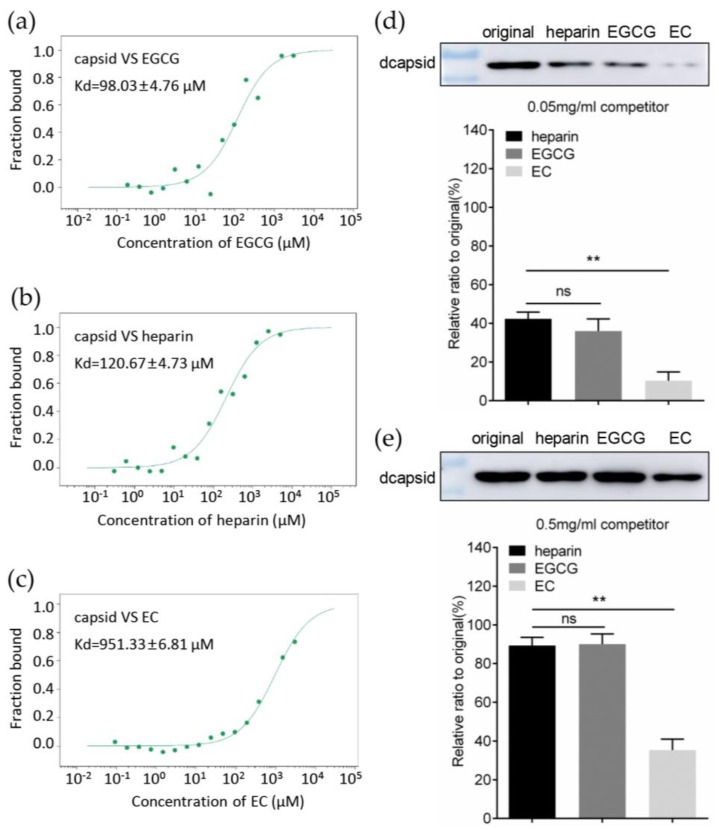
EGCG competitively inhibited capsid binding to heparan sulfate. (**a**–**c**) EGCG could interact with capsid or with heparin. The dcapsid protein was labeled with the Cy5 fluorophore and incubated with two-fold serial dilutions of EGCG or heprarin (10–50 nM), and then the Kd values of the dcapsid were measured for EGCG (**a**), heparin (**b**), and EC (**c**) were measured by MST. (**d**,**e**) EGCG eluted dcapsid protein bound to a heparin column with similar efficiency as heparin. Heparin-Sepharose HP column was pre-loaded with dcapsid and eluted with 0.05 (**d**) or 0.5 (**e**) mg/mL soluble competitor reagents (heparin, EGCG and EC). The eluted samples were analyzed using SDS-PAGE and immunoblotting. Three independent experiments were conducted. The results are presented as Mean ± SD and compared using Student’s *t*-test (n.s., not significant; * *p* < 0.05; ** *p* < 0.01).

PCV2 virions attached to host cells via direct interaction between viral capsid protein and cell surface heparan sulfate [10]. Therefore, we hypothesized that EGCG might also bind to capsid, and competitively block the interaction between PCV2 capsid and HS. The microscale thermophoresis assay (MST) was utilized to evaluate the affinity between compounds and protein by measuring the dissociation constant (Kd). The Kd between dcapsid and EGCG was 98.03 ± 4.76 μM, indicating that EGCG could indeed bind to capsid (Figure 4a), and capsid’s affinity to EGCG was comparable to the affinity for heparin (an HS analog similar in structure to the sulfated domain of HS), or even a little stronger than the latter (Kd = 120.67 ± 4.73 μM) (Figure 4b). However, the EC, which did not exhibit the inhibitory activity against PCV2 infection, could also bind to capsid with a 10-fold lower affinity than EGCG (Kd = 951.33 ± 6.81 μM) (Figure 4c). These findings implied that the interaction between PCV2 capsid and HS might be hindered by EGCG. This was confirmed by heparin column chromatography assay. Briefly, the dcapsid protein was pre-loaded onto a heparin-Sepharose column, then eluted by the various competitor reagents. The results showed that approximately 40% protein fraction (42.33 ± 3.52%) could be eluted by the EGCG at a concentration of 0.05 mg/mL, similar to the eluted level of heparin (36.00 ± 6.25%), while in case of EC only 10.33 ± 4.50% protein sample was eluted from the heparin column (Figure 4d), which was significantly reduced compared to the level of EGCG or heparin competitor reagents. The eluted fraction was further increased when the concentration of competitor reached 0.5 mg/mL. In that case, the heparin and EGCG could elute 89.33 ± 4.16% and 90.00 ± 5.29% of total protein, respectively (Figure 4e), and the difference between then was still not significant. In summary, EGCG could directly interact with PCV2 capsid, competitively inhibiting the binding of latter to HS.

### 3.5. Identification of Key Amino Acids in PCV2 Capsid Contributing to the Interaction with EGCG

**Figure 5:**
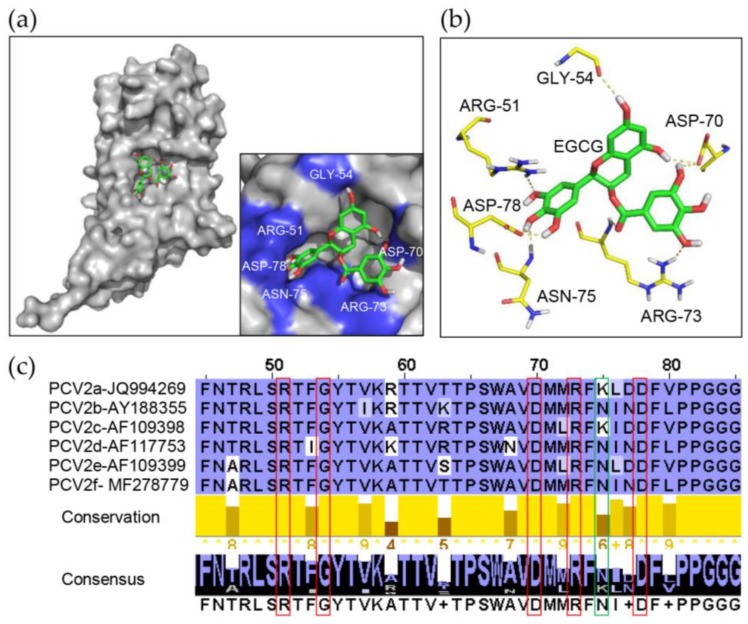
Molecular docking model of interaction between EGCG and PCV2 Capsid. (**a**) Structural diagram of EGCG-capsid complex based on the flexible docking model. The amino acids involved in the formation of the pocket accommodating EGCG molecule (green) were labeled blue in the dcapsid (light grey) in the zoom graph. (**b**) The putative amino acids (yellow) in dcapsid that could establish hydrogen bonds with EGCG (green) based on the docking model. The predicted hydrogen bonds are shown as a yellow dotted line. (**c**) The conservation of the key amino acids in the capsid to maintain the interaction among PCV2 subtypes. All the amino acid sequence data were obtained from GenBank. Among the predicted amino acids which might form hydrogen bonds with EGCG, the ones with high conservation (ARG-51, GLY 54, ASP-70, ARG-73 and ASP-78) are highlighted with a red frame, and the rest (ASN-75) are labeled with a green frame.

To further investigate the detailed structural character of the interaction between EGCG and capsid, the flexible docking model of the complex was established with AutoDock software 4.2 (Olson Lab, http://autodock.scripps.edu). The crystal structure of PCV2 dcapsid was obtained as PDB accession ID:3R0R and the whole domain of protein was docked with ligand. Several docking model structures were obtained, and the one with the lowest interface energy was adopted for further analysis, which showed that each EGCG molecule could be attached to a capsid monomer at a distinct pocket, which was completely exposed to the surface of PCV2 particles according to the revealed structure of the PCV2 VLPs [18], and the pocket was formed by some critical amino acids (Figure 5a), among which ARG-51, GLY 54, ASP-70, ARG-73, ASN-75, and ASP-78 were predicted to engage in hydrogen bonds with EGCG (Figure 5b). We established an amino acid sequence alignment to assess the conservation of these amino acid. It was demonstrated that all of these 6 amino acids were highly conserved in PCV2 ranging from PCV2a to PCV2f except for ASN-75 (Figure 5c), suggesting their critical importance for the interaction between capsid and EGCG molecule in the various subtypes of PCV2.

**Figure 6:**
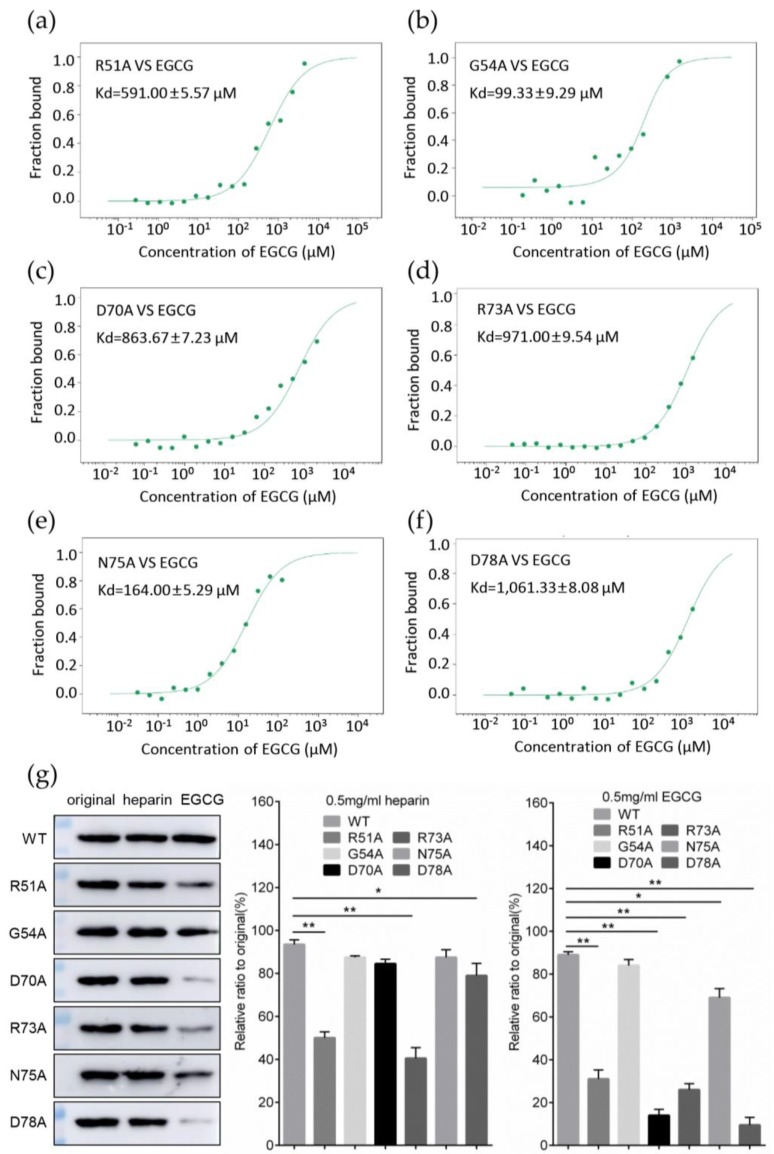
Verification of key amino acids involved in the interaction of EGCG with capsid. (**a**–**f**) The affinity impairment of capsid to EGCG induced by the key amino acid mutation. The Cy5 fluorophore labeled dcapsid mutants were incubated with two-fold serially dilutions of EGCG at room temperature for 15 min before being tested with MST, and the Kd values between EGCG and capsid mutants R51A (**a**), G54A (**b**), D70A (**c**), R73A (**d**), N75A (**e**) and D78A (**f**) were measured, respectively. (**g**) The eluted level of capsid mutants bound to the heparin column. Heparin column preloaded with dcapsid mutants was eluted with 0.5 mg/mL soluble heparin or EGCG. The eluted level of each mutants was analyzed using immunoblotting. Mean ± SD of three independent experiments were statistically analyzed using Student’s *t*-test (* *p* < 0.05; ** *p* < 0.01).

To test this hypothesis, and to identify the key amino acids for the interaction, we made a single-point mutation of these amino acids to alanine (A) residue and measured the affinity of each mutant to EGCG, the results of MST showed that the Kd of different mutants and EGCG could fluctuate significantly compared to the wild type (WT) capsid; among all the mutants, the D70A and D78A showed the loweest affinity to EGCG with Kd values of 863.67 ± 7.23 μM and 1061.33 ± 8.08 μM, respectively (Figure 6c,f), almost 10 times lower than the Kd of WT to EGCG (Kd = 98.03 ± 4.76 μM) (Figure 4a). Additionally, mutants aimed at arginine (R) residues also exhibited comparable reduction in affinity to EGCG, the Kd values of R51A and R73A were 591.00 ± 5.57 μM and 971.00 ± 9.54 μM, respectively (Figure 6a,d). On the contrary, the affinity of G54A and N75A mutants did not change significantly (Figure 6b,e). These results indicated that ARG-51, ARG-73, ASP-70 and ASP-78 were critical for the interaction of EGCG with the PCV2 capsid. To further assess the effect of these amino acids on the binding properties of capsid to HS, the heparin column chromatography assay of different mutants was performed and it showed that the eluted level of R51A, D70A, R73A and D78A mutants was remarkably reduced, with 0.5 mg/mL EGCG, especially in the case of D70A and D78A mutants, which is consistent with the results of Kd determination. Meanwhile, the mutation targeted to arginine residues (R51A and R73A) displayed the most significant eluted reduction with 0.5 mg/mL heparin (Figure 6g). Taken together, these results indicated that the ARG-51, ARG-73, ASP-70 and ASP-78 of PCV2 capsid protein were crucial for its interaction with EGCG, and the two arginine residues were also essential for binding with cell surface HS.

### 3.6. Replacement of Key Amino Acids in Capsid Weakens the Antiviral Effect of EGCG

**Figure 7:**
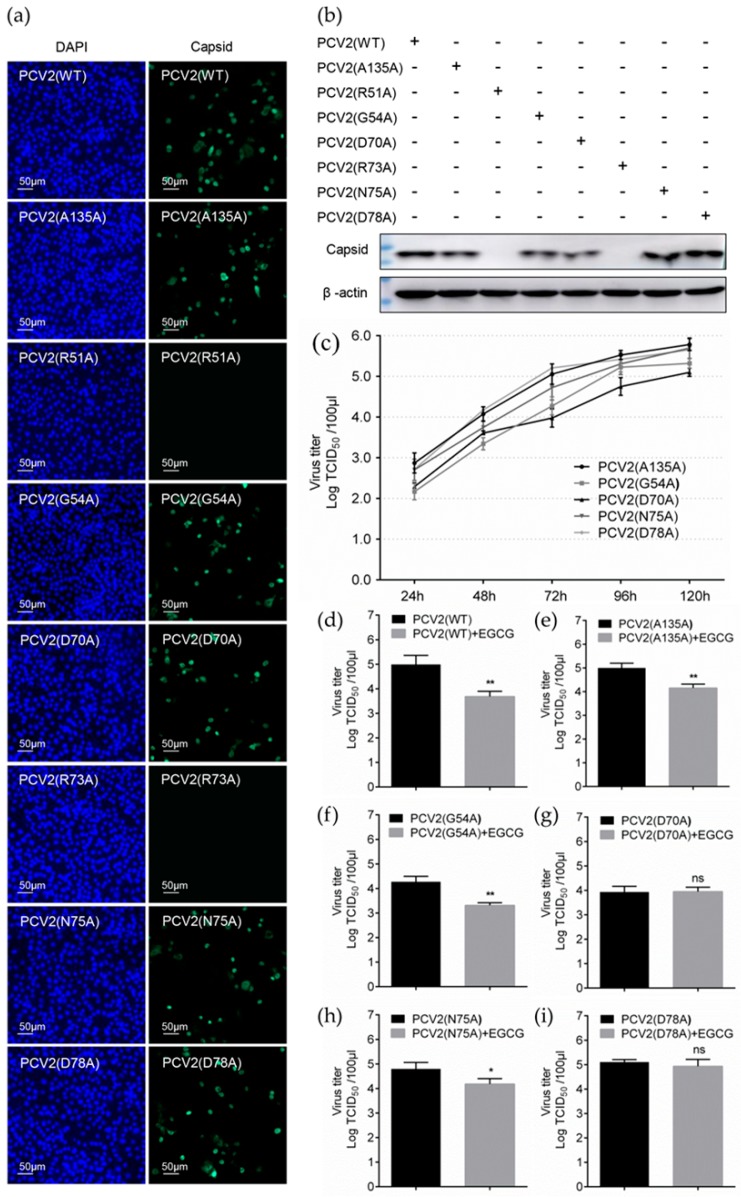
Regulation of EGCG antiviral activity by replacement of key amino acids in capsid. (**a**,**b**) The infectivity of rescued PCV2 mutants. The rescued PCV2 mutant virus was inoculated into PK-15 cells at MOI = 1.0; at 72 hpi the cell samples were detected by IFA (**a**), and the capsid expression was measured by immunoblotting (**b**). (**c**) One-step growth curve of PCV2 mutants. PK-15 cells were inoculated with mutant viruses at MOI = 1.0, TCID_50_ at various time points were determined by IFA and calculated according to the Reed-Muench method. (**d**–**i**) Antiviral effect of EGCG on PCV2 mutants. PK-15 cells were infected by mutant viruses at MOI = 1.0 with 100 μM EGCG treatment during the whole infective process and the titers were detected by IFA at 72 hpi. Mean ± SD of three independent experiments were statistically analyzed using Student’s *t*-test (* *p* < 0.05; ** *p* < 0.01).

To further confirm the role of the key amino acids in capsid towards the antiviral activity of EGCG, we tried to use virus rescue strategy to generate mutant viruses and evaluate the regulation in EGCG’s antiviral activity. The circularized PCV2 genome with R51A, G54A, D70A, R73A, N75A, and D78A mutations were transfected into PK-15 cells, respectively. Also, the genome with the A135A mutation was designated as a positive control. Transfected cells were serially passaged and cultured to harvest the rescued virus particles, which were then inoculated into PK-15 cells to test the infectivity. The results of IFA showed that PCV2 (G54A), PCV2 (D70A), PCV2 (N75A) and PCV2 (D78A) could be detectable at 72 hpi, similar to PCV2 (WT) and PCV2 (A135A) (Figure 7a). The results of immunoblotting also confirmed the capsid expression of these mutant viruses at 72 hpi (Figure 7b). One-step growth curves of PCV2 (G54A), PCV2 (D70A), PCV2 (N75A), and PCV2 (D78A) also displayed a similar replication ability to wild-type PCV2 (A135A) (Figure 7c). Surprisingly, PCV2 (G51A) and PCV2 (G73A) could not be rescued and obtained with the same operation (Figure 7a–c), which might be due to the replacement of these key arginine residues related to HS binding ability would cause the impairment of virus binding to cell surface, which eventually lead to the inability of virions to complete the infection process [18].

We next performed the infectivity assay of mutant viruses with 100 μM EGCG treatment during the whole infective process. The titers of mutant viruses were detected at 72 hpi and the results showed that EGCG could inhibit the infectivity of PCV2 (G54A) and PCV2 (N75A) (Figure 7f,h), similar to its antiviral effect on PCV2 (WT) and PCV2 (A135A) (Figure 7d,e). In contrast, the antiviral effect of EGCG could not be detected during the infection of PCV2 (D70A) and PCV2 (D78A) (Figure 7g,i), These findings are consistent with the results of affinity determinations (Figure 6c,f,g), indicating that ASP70 and ASP78 of capsid are essential for binding of PCV virions to EGCG.

## 4. Discussion

As the most prominent component of GTCs, EGCG displayed broad and distinguished anti-viral activity compared to other GTC members [36]. Here, we showed that EGCG exhibited an inhibitory effect on the PCV2 infection at the concentration of 100 μM, without causing significant toxicity to the host cells. EGCG acted directly on PCV2 virions rather than any cellular factors with or without the stimulation of virions, and EGCG inhibited virion attachment to host cells by competitively interacting with capsid with cell surface receptor, but it did not affect other post-binding stages.

Heparan sulfate is a linear, synthetic, acidic polysaccharide belonging to the glycosaminoglycan (GAG) family, which is widely distributed on the surface and in extracellular matrix of various mammalian cells [37]. HS was attached to the protein core and consisted of repeating 1 to 4 linked disaccharide units, one of which is an α-d-glucosamine residue and the other is an anionic acid. HS chains acquired negative charge though epimerization and terminal sulfation processes, thereby facilitating HS in establishing electrostatic interaction with positively charged motifs and residues [38], including signaling pathway proteins, growth factors, plasma proteins, immune modulators and viral structural proteins [39]. In general, these protein ligands possessed distinct HS binding region, which acted as a complementary structure for heparin-protein interactions [40]. Based on the known ligands’ structural details, the molecular modeling of protein-glycosaminoglycan revealed that HS binding regions formed a hydrophilic pocket wrapped around and folded over heparin oligosaccharides. The key factors to produce such hydrophilic pocket were the clusters contributed by basic amino acids, especially arginines and lysines [41,42].

The EGCG interacted with PCV2 capsid. Due to the considerable affinity, this interaction could competitively impair the binding of capsid to HS, thereby inhibiting the attachment of virions to the host cell surface. Meanwhile, the EC could not interact with the virus capsid, and hence did not exert an antiviral effect. Structurally, EGCG possessed numerous hydroxyl radicals attached on an extra benzene ring which is not the case for EC (Figure 1a), and the result of molecular docking showed that these additional free radicals were involved in forming hydrogen bonds with ASP70 and ARG-73 of capsid. Indeed, the mutagenesis assay demonstrated that aspartic acids (ASP70 and ASP78) and arginines (ARG51 and ARG73) were essential for EGCG- capsid interaction. It is also worth noticing that the arginine residues of GAG ligand were also important for the formation of a center with a high positive charge density, which could electrostatically interact with the acidic groups of HS; this was supported by the result of the replacement of ARG51 and ARG73 which remarkably attenuated the affinity of capsid to HS. Based on these findings, we conclude that the interaction of EGCG with PCV2 capsid might primarily depend on the formation of hydrogen bonds by specific positively charged amino acids in capsid, such as ARG51 and ARG73, while newly formed links might thus interfere with the original electrostatic interaction between capsid and HS, resulting in the impairment of virus attachment to the cell surface.

In previous studies, EGCG showed similar antiviral activity against various viruses, EGCG inhibited the infection of PRRSV with an EC_50_ of 48.2–63.09 μM in vitro [43], and EGCG exhibited an antiviral effect against JEV with an IC_50_ of 4.9–20 μM in vitro [44]. In our study, the EC_50_ of EGCG against PCV2 in vitro was calculated as 37.79 ± 1.64 μM. These comparable findings suggested that the antiviral activity of EGCG in vitro did not reach the efficacy of traditional antiviral drugs, such as ribavirin. It is worth noting that although this level of potency of EGCG at this stage is not of practical value in the treatment of clinical virus infections, the identification of key amino acids of viral proteins that bind EGCG is valuable for the development of structure-based anti-virus drugs, such as the EGCG-based synthetic EGCG palmitate, whose EC_50_ against PRRSV is decreased to 5.86–12.69 μM, which is nearly 5 times lower than for the original EGCG [45]. On the other hand, more research data focusing on in vivo antiviral effects of EGCG and detailed structural analysis of EGCG-protein interaction should be studied in future research to facilitate a more comprehensive assessment of the antiviral potential of EGCG.
